# Household Food Insecurity and Psychosocial Dysfunction in Ecuadorian Elementary Schoolchildren

**DOI:** 10.1155/2018/6067283

**Published:** 2018-08-13

**Authors:** M. Margaret Weigel, Rodrigo X. Armijos

**Affiliations:** ^1^Department of Environmental & Occupational Health, Indiana University-Bloomington School of Public Health, USA; ^2^Global Environmental Health Research Laboratory, Indiana University-Bloomington School of Public Health, USA

## Abstract

Household food insecurity (HFI) is a major global public health and pediatric concern due to its reported association with adverse child nutrition, growth, and health outcomes. Psychosocial dysfunction is a major cause of childhood disability. US and Canadian studies have linked HFI to poorer overall psychosocial dysfunction and specific dysfunction types in school-aged children, i.e., internalizing, externalizing, and attention behaviors. However, it is uncertain whether prior findings are generalizable to low- and middle-income country (LMIC) settings. We conducted a cross-sectional study to explore the association of HFI with psychosocial dysfunction in 6-12-year-old public elementary schoolchildren (n=279) residing in low-income neighborhoods in Quito, Ecuador. Maternal caregivers were interviewed to obtain data on child psychosocial dysfunction (Pediatric Symptom Checklist, PSC), food security (Household Food Security Survey Module), and maternal mental health (SF-36 Mental Composite Summary). Capillary blood samples were obtained from child participants to measure hemoglobin levels. The data were analyzed using general linear models with adjustment for covariates. The results revealed that HFI was associated with significantly higher overall average PSC scores (p=0.002) and with internalizing (p=0.001) and externalizing (p=0.03) but not attention subscale scores. However, anemia was independently associated with PSC attention subscale scores (p=0.015). This is the first study to report on the relationship between HFI and psychosocial dysfunction in school-aged children in a LMIC setting. It highlights the importance of improving policies and programs protecting vulnerable households from HFI. In addition to improving health and nutrition, such improvements could potentially reduce the burden of child psychosocial dysfunction.

## 1. Background

Household food insecurity (HFI) constitutes a major global public health and pediatric concern due to its high prevalence and association with adverse health and nutrition outcomes. Estimates from the 2014-2015 Gallup World Poll Survey suggest that 605 million or 41% of children under the age of 15 years reside in food insecure households worldwide, the majority of these being in low- and middle-income countries (LMIC) [1]. Household food insecurity is reported to be associated with adverse child nutrition, growth, and health outcomes [2–6]. In school-age children, it also has been linked to feelings of deprivation, shame [7], suboptimal academic performance, and poorer social skills [8]. The findings from a number of prior studies conducted in the US and Canada suggest that school-age children exposed to HFI have greater psychosocial dysfunction [9–16]. This is important since psychosocial dysfunction is a major cause of childhood disability that can adversely affect normal child development, social relations, academic performance, and quality of life. If not identified and treated early, it can persist into adolescence and later life to interfere with daily functioning and cause long-term work disability [17, 18].

Exposure to HFI can potentially promote psychosocial dysfunction in school-age children through both nutritional and nonnutritional pathways. For example, children in food insecure households tend to have poorer quality and less diverse diets, lower micronutrient blood levels, and anemia [2, 3]. In turn, anemia has been linked to poorer child cognitive, behavioral, and mental health outcomes [4, 19, 20]. Disruption of regular meal and other food-related routines also can cause stress and feeling of discomfort in children [11]. In addition, school-age children are often acutely aware of their household food security situation which may cause them to feel deprived, ashamed, or stigmatized [7]. Another potential pathway by which HFI can promote child psychosocial dysfunction is through its negative effects on maternal caregiver mental health and behavior [21]. For example, the inability to provide adequate food for their children and other household members may contribute to feelings of depression, anxiety, and stress [12, 22, 23]. This may reduce the ability of maternal caregivers to provide adequate levels of child stimulation and care leading to poorer child psychosocial functioning [21, 24].

Although findings from a number of previous studies generally agree that HFI is associated with poorer psychosocial functioning in US and Canadian school-aged children [9–16], disagreement exists with respect to the specific types of psychosocial dysfunction involved. For example, some [5, 9, 11, 14, 16] but not all studies [25, 26] linked HFI with greater internalizing behavior. Likewise, some authors reported that HFI is associated with increased externalizing/aggressive behaviors [5, 9, 11, 13, 26, 27] but several others did not [16, 25, 28]. While one study reported that schoolchildren living in food insecure homes tended to have greater attention/hyperactivity problems [25], another conducted in the same age group found no association [15].

The reason for these discrepancies among previous study findings is unclear. It is possible that they could be due to differences in the prevalence and severity of HFI and psychosocial dysfunction or in the poverty and other characteristics of the US and Canadian child samples. Methodological differences could also be responsible since the studies used different instruments to measure food security (e.g., 1-item or 2-item food sufficiency questions [11, 13, 25], USDA household food security module versions [5, 9, 26–28], and Community Childhood Hunger Identification Project measures [14–16]). Different instruments also were used by prior studies to assess psychosocial dysfunction such as the Pediatric Symptom Checklist [14, 15], Child Behavior Checklist [11, 15, 16, 25], Teacher's Report Form [15, 27], Social Skills Rating System [5, 25], and other measures [9, 13, 29]. Likewise, some of the discrepancies could be due to differences in study design, sample size, and unmeasured covariates in the statistical models used to analyze the data.

To the best of our knowledge, no studies have been published on the association of HFI with psychosocial dysfunction in school-aged children in Latin American or other LMIC populations. This is a major gap in the literature as it is uncertain whether the findings from studies conducted in high-income countries, in this case, the US and Canada, are generalizable to LMIC settings. This is important since these populations often differ regarding the context and prevalence of HFI, psychosocial disorders, and poverty as well as living conditions: family size, composition and dynamics, child malnutrition, access to governmental social safety net programs, and other characteristics. Such differences have the potential to influence the relationship between HFI and child psychosocial functioning.

We conducted a study in Ecuador, a Latin American LMIC, for the purpose of exploring whether exposure to HFI was associated with greater psychosocial dysfunction in 6-12-year-old children attending public elementary school in three low-income neighborhoods in Quito. Our working hypothesis was that children from food insecure compared to food secure households would exhibit greater overall psychosocial dysfunction and specific types of dysfunction, i.e., internalizing, externalizing, and attention behaviors. We hypothesized that this can occur through both nutritional and nonnutritional pathways. One possible pathway is through the adverse effects of HFI on dietary quality and diversity leading to lower blood hemoglobin levels and anemia in children. Another potential pathway involves the psychological stress generated by living under household conditions of food insecurity. Such stress can have a negative impact on the psychosocial functioning of children. In addition, the stress of living under conditions of food insecurity also may promote feelings of depression, anxiety, or other mental health disorders of maternal caregivers. This situation may reduce their ability to engage with and adequately care for their child.

## 2. Methods

### 2.1. Study Setting and Participants

The cross-sectional study was part of a larger investigation of the nutrition and health of low-income maternal caregivers and their school-age children. It was conducted in Quito, the capital city of Ecuador. Study data were collected during a three-month period (June-August, 2014). The participants were public elementary school children and their maternal caregivers who lived in three low-income neighborhoods: Cotocollao, El Camal, and Alangasi. The study team held initial meetings with administrators, teachers, parents, and their children in the three neighborhood elementary schools to discuss the study and determine their interest in participation. Children were eligible for possible inclusion if they were currently enrolled in one of the three schools in grades 1-6, they were between the ages of 6 and 12 years, and they and their maternal caregivers agreed they would participate. Maternal caregivers also had to meet certain inclusion criteria. Specifically, they had to currently reside in the same home as the child and be at least 18 years of age or older and not have any conditions that would make it difficult for them to understand or respond to the interview questions. From this pool, eligible maternal-child dyads were selected for study participation using a computerized random numbers table. The study protocol was approved by the institutional review boards of the University of Texas at El Paso and the Central University of Ecuador Biomedical Research Center. Participating maternal caregivers were required to give their written informed consent for themselves and their children and the children to give their informed assent, prior to the start of data collection. To prevent oversampling, only one maternal-child dyad from a household participated in the study.

Maternal caregivers were provided with oral and written interpretations of screening results from the present study as well as anthropometric, laboratory, and clinical indicators collected by the larger health and nutrition umbrella project. Where indicated or requested by maternal caregivers, the study team provided written referrals for follow-up care through the free Ecuadorian public health or the social security health systems. In addition, all child and maternal caregivers received a nutritious free breakfast provided by the study.

### 2.2. Outcome Variable

#### 2.2.1. Pediatric Symptom Checklist

The Pediatric Symptom Checklist (PSC), an instrument widely used in pediatric practice, educational settings, and research studies [30], was used to screen for child psychosocial dysfunction. The PSC contains 35 items that were scored by maternal caregivers as 0 (“never”), 1 (“sometimes”), or 2 (“often”). The total PSC score was calculated by summing the score for each item. Possible scores on the PSC ranged from 0 to 70. The higher the PSC score, the higher the overall level of psychosocial dysfunction [30].

The three subscales contained in the PSC were used to identify specific types of child psychosocial impairments: internalizing, externalizing, and attention [30]. The PSC internalizing subscale had five items suggestive of anxiety and/or depression with possible scores ranging from 0 to 10. The specific items included the following: whether the child feels sad, feels hopeless, is down on him/herself, worries a lot, and seems to be having less fun. The PSC externalizing subscale contained seven questions focused on conduct problems: fights with others, does not listen to rules, does not understand other people's feelings, teases others, blames others for his or her troubles, and takes things that do not belong to him/her, and refuses to share. Possible scores on this subscale ranged from 0 to 14. The PSC attention subscale had five items suggestive of hyperactivity including questions about the child acting fidgety, being unable to sit still, daydreaming too much, being distracted easily, having trouble concentrating, and acting as if driven by a motor. Possible scores on this subscale range from 0 to 10. On all three PSC subscales, the higher the score, the greater the degree of dysfunction [30].

The Spanish-language translation of the PSC^30^ used in the present study was previously confirmed as having good validity and reliability as a screening tool for psychosocial dysfunction in Latino children in the US [31–33], Chile [34], and Brazil [35] and is used in some Ecuadorian pediatric practice settings. In order to confirm the internal validity and reliability of the PSC for the local Ecuadorian child population, we performed Rasch model analysis, a type of Item Response Theory using WINSTEPS software (v. 3.91, Beaverton, OR). The findings from the analyses suggested that basic Rasch model assumptions were met. Briefly, the infit statistic scores for the 35 individual PSC items ranged from 0.75 to 1.4 indicating they were within the 0.6-1.4 range of values considered acceptable for an adequate model fit [36, 37]. The mean item-infit score for the overall instrument was 1.02 with a standardized score of 0.0 suggesting a good unidimensional construct. The separation statistics analysis showed that overall Rasch reliability statistic (analogus to Cronbach's alpha or KR-20) also was adequate, i.e., 0.82 [37].

#### 2.2.2. Household Food Security

Data on household food security status were collected from the maternal caregiver participants using an adapted Spanish-language version of the USDA-ERS Household Food Security Survey Module (HFSSM) previously validated for the same urban Ecuadorian population [23]. The experience-based food security instrument contained 18 questions which ranged from worrying about running out of food to child food deprivation. Affirmative responses to the 18 items were added to construct the raw score. As in the original HFSSM, child households with raw scores totaling 0-2 were classified as food secure while those with scores ≥ 3 were classified as food insecure [23, 29].

### 2.3. Covariates

#### 2.3.1. Sociodemographic Characteristics

Face-to-face interviews conducted with maternal caregivers were used to collect data on child, maternal, and household sociodemographic characteristics. Data collected on child characteristics included age, gender, and ethnicity. Maternal caregiver attributes included age, years of formal education, marital status, and occupation. The information collected on household characteristics included neighborhood location, household size, and number of minor children living in the home, monthly* per capita* income, and household participation in social safety net programs.

#### 2.3.2. Maternal Mental Health

The Mental Composite Summary (MCS) score from the SF-36 (Spanish version) was used to assess maternal mental health status [38]. The MCS subscale included questions on vitality (4 items), social functioning (2 items), role limitations due to emotional problems (3 items), and emotional well-being (5 items). Higher scores represent better mental health. The validity and reliability of the SF-36 Spanish version instrument were previously validated for urban Ecuadorian women [39] and Ecuadorian immigrants [40].

#### 2.3.3. Child Blood Hemoglobin

Capillary blood samples obtained by sterile fingerstick were used to measure child hemoglobin levels. These were analyzed using the HemoCue photometer (Model 201, HemoCue Inc., CA). Established hemoglobin concentration cutoff-points were used to identify 6-12-year-old children with anemia defined as < 110 g/L at sea level [41]. Hemoglobin values were subsequently adjusted for altitude as the Quito neighborhoods in which the child participants lived ranged from 2613 to 2812 meters above sea level. These adjustments were from -15 g/L to -19 g/L depending on altitude of the child residence [41].

### 2.4. Data Analysis

No statistically significant differences were detected among the three public elementary school sites with respect to household food security status, child PSC and PSC subscale scores, household income, or other child, maternal, or household characteristics. Thus, these were subsequently combined for the data analyses. Summary statistics are presented as number (%) or mean ± SD. The association of household food security status with average PSC scores and scores from the three PSC subscales was explored using general linear models (GLM). The GLM analysis results are presented in the tables and text as unadjusted and adjusted means. The model covariates included child age (years) and sex (female vs. male), child hemoglobin status (anemia vs. no anemia), maternal caregiver marital status (legally married vs. other), maternal caregiver occupation (full-time stay-at-home mother vs. other), maternal MCS score, monthly* per capita* income (in US dollars), and number of minor children in the home. In this study, values < 0.05 were considered statistically significant.

## 3. Results

The prevalence of food insecurity reported by maternal caregivers was high, affecting 78% of the 279 study households.  Table 1 displays the sociodemographic characteristics of the child and maternal caregiver participants and their households. The average age of the child participants was nine years, slightly more than half were girls, and 93% belonged to the mestizo majority ethnic group. The blood hemoglobin measurements of one-third of child participants indicated that they suffered from anemia. Most maternal caregivers were in their mid-30s and had around eight years of formal schooling. Nearly two-thirds indicated that they were legally married and 44% reported that they were full-time, stay-at-home mothers. The rest worked in full- or part-time jobs outside the home. Household size averaged nearly five members. The number of minor children in the household was around two. The* per capita* income of households averaged slightly under $130/month but despite their low incomes, fewer than 10% reported that they currently received any governmental or nongovernmental social safety net benefits. None of these characteristics of the children, maternal caretakers, and households were found to be associated with HFI (data not shown).

Table 2 shows the findings from the unadjusted and adjusted GLM analyses investigating the relationship between household food security status and average PSC total and subscale scores. As Table 2 shows, the unadjusted analysis results indicated that average total PSC scores for children from food insecure households were significantly increased compared to their food secure counterparts. Likewise, those from food insecure versus food secure households had higher average PSC internalizing and externalizing subscale scores but not attention subscale scores.

Several child and maternal caregiver variables were also associated with average overall PSC and PSC subscale scores. For example, child age was positively associated with internalizing (p=0.001) and negatively associated with attention (p=0.026) subscale scores. Female gender was marginally but positively associated with internalizing subscale scores (p=0.05). Positive associations also were identified between child anemia and overall PSC scores (p=0.049) as well as attention (p=0.015) subscale scores. Maternal MCS scores were positively associated with child PSC scores (p=0.0001) and internalizing (p=0.0001) and externalizing (p=0.001) subscale scores. Finally, having a full-time stay-at-home mother was inversely associated with overall child PSC scores (p=0.004) as well as internalizing (p=0.021) and externalizing (p=0.019) subscale scores. However, none of the other child, maternal, or household variables measured in the study were associated with average overall PSC or PSC subscale scores.

The results of the adjusted GLM analyses, shown in Table 2, also indicated that HFI was independently associated with higher average overall PSC scores and internalizing and externalizing subscale scores, after adjusting for covariates including child (gender, age, and ethnicity), maternal (full-time, stay-at-home mom status, and MCS score), and household characteristics (number of minor children living in home, monthly* per capita* income).

However, similar to the unadjusted analysis results, no significant association was identified in the covariate adjusted analysis between HFI and child PCS attention subscale scores.

## 4. Discussion

To the best of our knowledge, the present study is the first to report on the relationship of household food insecurity (HFI) with child psychosocial dysfunction in a Latin American or other LMIC population context. The high (78%) prevalence of food insecurity reported by study households is consistent with previously published estimates for other Ecuadorian groups [39, 42, 43]. The study data support our hypothesis that compared to school-age children living in food secure homes, those from food insecure households suffer from greater psychosocial dysfunction as reflected by their higher average scores on the PSC and PSC internalizing and externalizing subscales. This finding is consistent with the literature on US and Canadian children reporting similar positive associations for overall psychosocial dysfunction [9–16] as well as internalizing [5, 9, 11, 14, 16] and externalizing or aggressive behaviors [5, 9, 11, 13, 26, 27].

Different from our hypothesis, the study data did not support the supposition that children from food insecure homes would have greater hyperactivity as indicated by their PSC attention subscale scores. However, it is possible that HFI could still be indirectly involved since child participants suffering from anemia had higher average attention scores suggestive of greater hyperactivity. Although no statistically significant differences were identified between HFI and anemia in the child participants, anemia is a well-documented risk factor for poorer behavioral and mental health outcomes [4, 19, 20]. HFI has been reported as associated with lower dietary intakes of iron-rich foods and other micronutrients in Ecuadorian households [23], one cause of anemia. Another possible cause could be intestinal infections associated with consumption of unsafe food and beverages. Household food insecurity has been previously linked to gastrointestinal and other types of infections in children and adults [39, 44, 45]. In any case, whether the cause is dietary, infection, or both improving child access to safe and nutritious food including items fortified with iron and other micronutrients could potentially improve child psychosocial functioning in the area of attention behavior.

This exploratory study has several potential limitations. For example, the cross-sectional study design allows for inference but does not permit establishment of temporal or causal effects. While HFI may increase the risk for poorer child psychosocial or maternal mental health through nutritional, psychosocial, or other pathways, it is also possible that the relationship is bidirectional whereby these factors may also promote food insecurity. Another potential limitation is that since the measurement of food security was restricted to the previous 12-month period, it might not reflect household food situations over a longer period of time. In addition, since children are often protected from food insecurity by adults, it is possible that the food security situation reported by the maternal caregivers for their households may not necessarily reflect that of their child. It is also pointed out that maternal caregivers were the source of information regarding both their child's psychosocial dysfunction (PSC) as well as their own mental health status (SF-36 MCS) so reporting bias could have influenced the study results. Maternal caregivers who suffer from depression, anxiety, or other mental health issues might be more likely to perceive and report greater levels of psychosocial and learning problems in their children than others. For that reason, we suggest that future studies use teacher or clinician ratings to supplement the psychosocial assessments made by parental caregivers. Finally, although we adjusted for poverty-related variables, child characteristics, maternal mental health, and other characteristics, it is possible that confounding could have occurred due to unmeasured variables.

Despite these potential limitations, our study makes an important contribution to the literature linking household food insecurity with psychosocial dysfunction in school-age children and extending it to a LMIC population setting for the first time. Food insecurity is a potentially modifiable household condition. The study findings highlight the importance of improving social and economic policies and programs that protect vulnerable children and their families from the experience and adverse consequences of household food insecurity.

## Figures and Tables

**Table 1:** Sample sociodemographic characteristics (n=279).

	**Mean ± SD ** **or ** **No. (%)**
**Child Characteristics**	

Age (yrs)	9.0 ± 1.9

6	31 (11.1)

7	39 (14.0)

8	43 (15.4)

9	47 (16.8)

10	52 (18.6)

11	33 (11.8)

12	34 (12.2)

Gender (female)	155 (55.6)

Ethnicity (Mestizo)	258 (92.5)

Anemia	91 (32.6)

**Maternal Characteristics**	

Age (yrs)	35.6 ± 8.1

Formal education (yrs completed)	7.8 ± 3.8

Marital status (legally married)	176 (63.1)

Full-time stay-at-home mother	124 (44.4)

SF-36 MCS score	67.0 ± 14

**Household Characteristics**	

Household participation in any social safety net programs	20 (7.2)

Monthly per capita household income (U.S.$)	127 ± 91

Household size (members)	4.7 ± 1.4

No. minor children in home	2.3 ± 0.9

Neighborhoods: El Camal	102 (36.6)

Cotocollao	114 (40.9)

Alangasi	63 (22.6)

**Table 2:** Association of HFI with overall PSC and internalizing, externalizing, and attention subscale scores.

	All (n=279)	Food secure (n=61)		Food insecure (n=218)			
	Mean ± S.D.	Mean ± S.D.	Adjusted mean*∗*	Mean ± S.D.	Adjusted mean*∗*	GLM unadjusted model p	GLM adjusted model p
**Overall PSC score **	21.9 ± 9.0	18.4 ± 7.2	18.6	22.9 ± 9.3	22.8	0.001	0.001

**Subscale Scores**							

Internalizing	2.3 ± 2.0	1.5 ± 1.5	1.6	2.5 ± 2.0	2.4	0.001	0.001

Externalizing	3.5 ± 2.9	2.5 ± 2.6	2.6	3.8 ± 2.9	3.8	0.002	0.03

Attention	6.5 ± 2.1	6.2 ± 1.9	6.1	6.6 ± 2.1	6.6	0.15	0.72

*∗* Analyses adjusted for child gender (female), child age (years), ethnicity, child anemia, having a full-time stay-at-home mother, maternal SF-36 Mental Composite Summary (MCS) score, number of minor children living in home, and household monthly *per capita* income.

## Data Availability

The data used to support the findings of this study are available from the corresponding author upon reasonable request.
